# Early Indications of Insanity

**Published:** 1879-04

**Authors:** Judson B. Andrews

**Affiliations:** Assistant Physician, New York State Lunatic Asylum; Utica, N. Y.


*ART. III.—Early Indications of Insanity.* An Address delivered before the Alumni Association of the Buffalo Medical College, February 23d, 1879. By JUDSON B. ANDREWS, M. D., Assistant Physician, New York State Lunatic Asylum.

I desire to acknowledge the honor of being asked to address the Alumni of the Buffalo Medical College on the Anniversary occasion. There are gathered here those, who for years, have been our leaders and instructors in medical science; those who have gained wisdom and experience by lives devoted to the practice of medicine; those who now go forth to lives of usefulness, and let us hope to fortune, in their chosen field of labor; and others whose presence here to-night tells the interest and kindly feeling they entertain toward the College and the profession. To address such an audience carries with it a responsibility one might well hesitate to assume, but, having assumed it, it only remains to bespeak your indulgent consideration of a few remarks upon the subject of insanity, and the early indications of the disease.

As sanity, or the possession of reason in normal, healthful activity, is the most valued of God's gifts to man, so insanity, or the perversion or impairment of reason, through disease, is a loss not easily estimated. Other forms of disease affect the individual, mainly in his relation to the physical world. However extensive the injury to the body, however helpless the person may become, the intellectual power, and the moral responsibility, may still be left in undiminished strength. Insanity, on the other hand, involves the higher life of the individual; it affects his relations to his fellow-man, and impairs or destroys his self-control, and therefore his responsibility before the law, and may render him a hopeless wreck, irresponsible, and entirely dependent upon others.

The study of insanity has been under difficulties and obstacles, more numerous and serious, than have surrounded investigations in other departments of medicine. A potent reason is found in the fact, that a symptom of the disease, the mental aberration, was, for a long time, mistaken for the disease itself. This brought most prominently into view the mental and moral phenomena, and these were studied to the exclusion of the real disease. Investigations in this direction naturally fell into the hands of metaphysicians and theologians, by whom insanity was thought to be a disease of the mind or of the moral nature, and as the latter, to be synonymous with sin. Speculations of this character accomplished little for science, and still less for the insane. The treatment founded on these theories, consisted of amusements, mental exercises as a recreation, and of appeals to conscience, or to reason which are already involved. There was, in this, only mystery to the common mind, and to the professional mind nothing tangible or satisfactory, and it was only when the real nature of insanity was appreciated, that the light of truth dispelled the darkness of former superstition and error. The recognition of the theory that the brain is the instrument of the mind, and that perverted mental manifestations are due to disease of that organ, was the great step of progress in the study of insanity. Hitherto the subject had been approached from the wrong direction, but now there was in the disease of the physical organism a material something to account for the facts observed; something to investigate; something for the physician to do. For the first time the disease was brought with the purview of rational medicine, and the result has been the steady increase of knowledge of the disease and of its treatment.

As a general statement of fact, the cause of all insanity, as of other diseases, is to be found in the neglect, or in the direct or incidental infraction of the established laws of physical or mental health. It may originate from the physical side of man's nature, as from some form of ill-health, in which the nutrition of the brain in affected, or from the mental side, when excessive brain action produces the same result. In the ambition to become rich, to acquire power, to gain prominence in science or literature, the plainest laws of existence are too often transgressed. The claim of the body, especially the brain, for repose, for sleep, or even for nourishment to repair the waste of ordinary or excessive activity, is disregarded, and the often vain hope is fostered that an escape from the penalty of our actions is not only possible, but certain. The folly of this course has been plainly pointed out by a recent writer,* in the following well chosen words: "This marvelously constituted brain—men may ignore it—despise it—degrade it—but they do it at their peril; and let them remember that whether the result is or is not actual madness, they will pay the penalty sooner or later, in some form or other; they will not be permitted to escape the consequence of the infraction of the laws on which its integrity hangs."

A powerful predisposing cause of insanity is found in hereditary tendency. This is sometimes sufficient to account for its presence, under the operation of comparatively slight exciting causes. This is but stating a well known principle of medical science, and one fully recognized. It is impossible to estimate the force of heredity in the production of disease of the brain, but that it in-

* Insanity and its Prevention. Dr. Daniel Hack Tuke.

creases the liability to attacks, is beyond cavil or dispute. The instability of nerve element, implied in heredity, has a positive influence, and is a definite power.

In judging of insanity, it is important to have clearly in mind what it really is, what changes it produces in the mental and moral status of the individual. Practically, insanity is shown by a change in the disposition, sentiments, desires, habits, conduct or opinions, induced by, and founded upon disordered states of the brain. Every case is to be judged not by any arbitary standard, but by the change in the person himself. Each one, therefore, becomes the measure of himself, and we are to inquire what the individual was, and what he has become through disordered conditions of the brain. When viewed from this stand-point there is nothing of mystery in the disease; nothing to separate it from the field of general medicine, or to prevent its being investigated by all.

"There is nothing mysterious or peculiar in the methods of study or treatment. It is the patient and careful investigation of laws, and the application of well recognized principles in medical science; and not a question of interpretation of mental phenomena, or the study of mind, so much as an observation of the reciprocal influence of morbid physical and psychical states on the great nervous center, the brain." *

Its investigation is specially important to the general practitioner, as upon him devolves the responsibility of the diagnosis, and of the treatment of the disease in its initiatory stages. He is also made by legal enactment competent to decide the question of its existence, and of the necessity of care in a hospital.

The early indications of insanity assume an importance as they mark the period when treatment may be of avail in averting a threatened attack. The pathologic processes in the brain which produce insanity are preceded by certain changes in the physical system and in the mental habits of the individual, which, if understood and appreciated, are often susceptible of arrest by proper hygienic and therapeutic measures. It, however, sometimes hap-

---

* "Insanity and its Relation to Medicine." By Dr. John P. Gray. President's Address. Transactions of New York State Medical Society for 1868, page 86.

pens that the aid of the physician is not invoked till the disease has openly declared itself. This arises from the fact that by the great majority of individuals the occurrence of insanity, in their own persons or families, is not considered as within the bounds of probability, and all the initiatory conditions are overlooked or disregarded. Again, the changes are of such an insidious character, or are accounted for so fully by existing circumstances, that the patient and friends are entirely misled as to their real import.

Among the earliest indications which should attract attention as a precursor of insanity is the occurrence of morbid dreams. The mental activity during sleep, which constitutes dreaming, is so common in the experience of most people as not to be considered important as an indication of disease. There are, however, many cases on record in which dreams have preceded insanity, as well as other disordered states. Dreams, especially of a frightful character, as of being tortured or murdered, of dying or of the grave, occurring in persons unaccustomed to dreaming, and without a definite exciting cause, are often prodromic of disorder of the brain and of the nervous system, and have long been recognized by medical writers as worthy of consideration. "Such symptoms in medical psychology," says Tuke, "are like the delicate clouds in the sky in meteorology; they indicate to the practised observer the coming storm."

Of still more importance, and also of greater frequency as a precursor of insanity, is impairment of the function of sleep:

> "Sleep, that knits up the ravell'd sleave of care,
> The death of each day's life, sore labour's bath,
> Balm of hurt minds, great nature's second course,
> Chief nourisher in life's feast."

This impairment may vary from a simple state of wakefulness to one of persistent sleeplessness. In a normal healthy condition sleep naturally follows activity; action and repose being the physiological sequence. In disordered states of the brain the operation of this general law is often interrupted. Action is often but the stimulus or spur to increase activity. From disturbance of the circulation within the cranium there results an irritability of nerve tissue which may prolong the existing cerebral excitement. In this state thoughts follow each other in quick succession; past events of life are recalled with painful minuteness, or plans for the future present themselves in all the varied forms and combinations of which the imagination is capable; or, again, the most trivial matters demand and enforce the attention, in spite of the strongest efforts of the will to seek repose, and to bound the mental horizon with the one thought of sleep. At last, after many vain efforts, and perhaps from sheer exhaustion, sleep visits the weary one. It is but a fitful slumber which yields to ready waking, and the morning light finds the sufferer unrefreshed and unfitted for the labor of the day. Such states frequently arise from over-work, either mental or physical, and when the former is popularly called "mental strains." The liability to this is greatest among those engaged in public life, or among professional or business men, or those at the head of large corporations. Instances will occur to all in which wakefulness or entire loss of sleep has been thus induced. It does not always lead to insanity, but is, in all cases, a warning of danger which can not with safety be disregarded.

Impairment of sleep may result from exceptional causes—the accidents of life rather than the daily habit. Such are an additional and unusual amount of labor, unexpectedly thrown upon the individual, from which he can not escape; or it may be the overpowering influence of some emotion, as a long period of anxiety over the illness of a dear friend, or perhaps grief at his death, or the worry that comes from the sudden destruction of one's hopes or prospects in life. Again there may be no mental or physical cause apparent, sufficient to account for the disturbance of sleep, but this seems to be the earliest of a series of conditions which may gradually culminate in insanity. This statement, however, simply shows the imperfection and incompleteness of our knowledge, as it is impossible to accept the belief that an effect can be produced without an adequate exciting cause.

Disturbances of other functions than that of sleep are common as precursors of insanity. The following combination of symptoms, when not otherwise fully explained, should receive attention as indicating the danger which threatens. There is loss of appetite and indigestion, with pain, eructation, flatulence, heart burn, and offensive breath. The circulation is disturbed, the heart beating irregularly, or with diminished force, and the pulse is increased or lowered in frequency. There are flashes of heat, alternating with chilliness, and coldness of the extremities. The skin is harsh and dry; the bowels are constipated; there is usually loss of flesh which may extend to emaciation, and the whole organism sympathizes in the general depression.

There is another array of symptoms, in part the opposite of that described, which also frequently precedes an attack of insanity, especially when it is rapid in its access. The heart's action is increased in force, the pulse is full and strong, the face flushed, the eyes injected, and the temperature of the surface slightly elevated. The appetite may be unaffected, or even ravenous, but there is usually loss of flesh. In this condition there is marked increase in the activity of the vital functions, manifested more especially in the circulatory system. This combination of symptoms may furnish a warning of approaching disorder of the brain, usually of a congestive or hyperæmic character. It is, however, so frequently found in the early stages of febrile disease, that its real significance as an indication of insanity, may be overlooked or misinterpreted.

In other cases there may exist no such association of morbid symptoms, which forces itself upon the attention, and so often points to the brain as the organ directly threatened. There may be disease in some other part of the system, by which the nerve centers may become implicated, as shown by the abnormal mental action. The heart may fail to supply the amount of blood necessary for the nutrition of the brain, or the arteries and capillaries to distribute the life giving fluid to its delicate tissues. The lungs may be interrupted in the performance of their duty to supply the purifying and exhilarating oxygen, or the glandular system may be disturbed in its offices of secretion or elimination.

Disturbances of sensation not only exist prior to attacks of insanity, but often continue as serious symptoms of the disease. Among the most frequent of these is headache. This is usually described as a dull, aching sensation, but sometimes as a sharp, acute pain. It is often accompanied by a feeling as if a band was drawn tightly about the head, or as if the hat was too small; or, again, there is a sense of pressure, as if the brain was too large for the skull. Some complain of a throbbing of the arteries, others of a singing or a roaring in the ears, or a noise of falling water, much the same as that produced by quinine, when taken in full doses. In other cases there is a feeling of excessive heat, or of a crackling or crepitant sensation, often likened to the sound made by frying. The last mentioned sensations are more often referred to the vertex, and occur in the nervous and excitable, especially in women. The pain in the head is located most frequently in the frontal and temporal regions, and next in order in the occipital, and is produced by disturbances in the circulation of the blood in the meninges. These abnormal sensations are frequently followed by wakefulness or entire loss of sleep.

Muscular restlessness is often present, and may be manifested either in localized movements of the extremities or in the more general movements of the whole body. The hands or feet may be kept almost constantly occupied during the waking hours in the repetition of certain motions, while the abstraction of the individual gives them the appearance of being unconsciously or automatically performed. Again the person may move restlessly about without apparent purpose or design, and without being able to assign any cause, further than an inexplicable sense of uneasiness or discomfort. This condition is dependent upon nervous irritability, in turn due to some enfeebling mental or physical cause. Another state, the opposite of this, sometimes exists, in which there is not only a disinclination to muscular movements, but action, both mental and bodily, is performed only by a strong effort and the direct exercise of the will. The feeling of weariness may be insupportable, and the tendency to sleep yielded to upon the most inopportune occasions. The loss of control over muscular movements, indicated in these conditions, and that shown, though in less degree, in the irregular twitching or jerking of the muscles of the tongue, and of those about the mouth, sometimes become important aids in the early diagnosis of insanity.

With these changes in the physical system, and preceding the outbreak of the disease, there are necessarily associated disturbances of the mental state. The earliest are commonly found on the side of the emotions. These may be slight, amounting only to an exaggeration of the ordinary emotional status; or they may be more marked, and the inclination to laugh or to cry many be yielded to in spite of efforts to control the feelings. There may be no adequate cause for the unusual emotional excitement, or, if such exists, the exhibition of it may be contrary to the habit of the individual. This disturbance of the emotions frequently takes the form of a feeling of depression. There may be loss of spirits, or a shading off from the natural cheerfulness of disposition, noticeable to friends, and at first fully appreciated by the person himself. When rallied a smile may, for a moment, light up the countenance, and a cheerful remark may, for an instant, dispel the gloom, but a relapse soon follows. A cloud seem settling down, which grows darker as it approaches nearer. The despondency deepens, till that state is reached,

> "When nature, being oppress'd commands the mind
> To suffer with the body."

There are forebodings of some indefinite, indefinable evil impending, from which no way of escape lies open. Then commences an introspection; all the thoughts are turned inwards; all the acts of life are subject to a review and scrutiny, more severe than by the most unfriendly tribunal. The judgment rendered is always adverse and self-convicting. Notwithstanding the verdict is declared against himself, the unhappy man only returns to the task, and repeats the process.

In the direction of depression, and partaking of the same general character, is the development of scruples of conscience. These may exist concerning the performance of duty toward God or toward man. They sometimes lead to an unhappy zeal in the observance of religious rites and ceremonies, and to self accusation of intentional wrong, if not of criminal action, in the simplest affairs of life. This occurs alike to those of the most exemplary lives, and of undoubted Christian faith, and to the wicked and depraved, and is often considered only an evidence of a tender conscience. In this state many a one whose life has been a model of honest purpose and faithful action, accepts the inability to lament over his conduct, as an evidence of hypocrisy and hardness of heart. He seizes upon some trifling act which has nothing of the moral element in it, raises it to a position of vital importance, and makes to cluster about it the whole issue of life in this world and the next. It is not a matter of surprise that when this condition is not recognised as dependent upon disease of the physical organism, the spiritual aid of the priest should be invoked, rather than the more material support of the physician, or that insanity should be considered as a disease of the spiritual nature. Dr. Tuke says upon this point, "sudden and extraordinary scruples should also excite attention and concern, indicating as they frequently do, latent brain mischief, and a twist in the emotions which, although seemingly leaning to virtue's side, may nevertheless be pure delusions, simulating conscientious duty, and leading the misguided fancy into courses of action which, if the brain fog is ever dispelled by the sun of returning health, are remembered with bitter but unavailing regret."

Another indication of commencing insanity may be manifested in a change of conduct, in the direction of an unnatural indecision of purpose. This may occur regarding matters of little real moment as well as those of vital importance. The clothing to be worn, or the food to be eaten, or any of the minor affairs of every day life, which are ordinarily decided without thought or question, then become subjects of serious consideration, and are magnified to a degree out of all proportion to their significance. Other questions which demand discrimination and a just comprehension of causes and effects, produce an agony of doubt, which obscures the judgment and prevents intelligent action. Whatever course is finally resolved upon, may be given up for another, which in time makes place for some new line of conduct. The vacillation and uncertainty thus exhibited, may be in strong contrast to the ordinary stability of purpose and character.

A change in disposition, without sufficient rational cause, may have the same significance. The naturally gentle and amiable may become irritable, morose or fault finding. This change is manifested, first, toward the members of the family and intimate associates, and is concealed from ordinary or casual acquaintances. It, however, gains more complete control, and affects the individual in all the relations of life. Unfounded suspicions and jealousies, in the beginning vague and shadowy, give rise to a belief in the existence of a general spirit of opposition to the plans and purposes in which the person is interested. At first they are reluctantly expressed, and readily removed by appeals to reason. Eventually they are attached to individuals, and becoming more fixed in the mind, often take the form of hatred and opposition to the most loved and nearest of kin or to the dearest friends.

An unnatural buoyancy of feeling or levity of manner, in contrast to former habit and disposition, sometimes indicates the departure from the normal mental state. Friends are apt to ignore this condition, as it seems to them to be of good omen. "It is a symptom serious in itself, but of more decided import when attended by alternations of depression." Other early changes may also show increased mental activity, which takes the form of exaltation or exaggeration. Under the influence of this the quiet and retiring become talkative and obtrusive, the dependent and modest, self-reliant and self-confident. In daily life this leads the business man to entertain an extravagant view of his ability, to overestimate his financial strength, to talk boastingly of his past success, or of his prospects, and still further to enter upon schemes for the acquirement of wealth, which are unwarranted, injudicious, and often ruinous. It makes the economical housewife wasteful in expenditures, or to neglect home duties for unusual excitements of social life, while the youth under the same influence, suddenly acquires, in his own estimation, the mental stature of manhood, and all attempts to control or restrain only provoke opposition, or open conflict with proper authority. Regarding the diagnostic value of this state of increased mental activity, "it is most important to remember that exuberant spirits, mental exhilaration, loquacity, when unusual to the individual, are fully as serious indications as the opposite state of mind."

Other changes worthy of note, though less important than those already mentioned, are departures from neatness and regard for dress and personal appearance, to a neglect or disregard of them; or, on the contrary, there may be developed a love for show and externals in matters of apparel, entirely inconsistent with previous habits.

There are instances in which insanity is so insidious in its approach that it is not recognized until fully established, and sometimes not until it has already reached a chronic stage. In these cases there is often an intensification of natural characteristics; this occurs especially in persons of marked peculiarities of temper or of disposition, which often exist in those of a congenitally feeble mental organization, or, on the other hand, in those possessing mental strength and vigor, but who are without proper balance or training. It is sometimes difficult in these instances to distinguish the changes wrought by disease from capriciousness, eccentricity or false views of life. Such people are inclined to be irritable, quarrelsome, self-willed, and are unpleasant in their families, and in their relations as neighbors. They imagine or greatly exaggerate grievances, and are given to the habit of trying to rectify them by appeals to law. They continue to grow more ill-tempered and irascible, till they sometimes become the terror of a community. At last, from the violence of their conduct, they may be brought to the notice of the authorities, when a thorough examination reveals their actual mental condition, and they are shown to be insane and irresponsible.

I have thus attempted to sketch briefly and concisely the most common and prominent of the initiatory changes, both physical and mental, which precede and point to an approaching attack of insanity, and also to notice some of the circumstances which may obscure the early recognition of the disease. It is not claimed that the list is complete, or that the various combinations in which the symptoms exist have been presented. These will differ much, as do the history of the cases under investigation. They will, however, be found to be naturally classified under the divisions employed—disturbances of function, of sensation, and of mental states. The manifestations of the mental disturbance, both in subjects and direction, present the greatest diversity, and are as various as the ages, the educational advantages, the social circumstances and surroundings of individuals, or the troubles or misfortunes with which their lives may be checkered.

If, in some of the descriptions of mental changes, the boundary line of sanity has been passed, it must be borne in mind that the border land between sanity and insanity is sometimes shadowy and indistinct, that it may be crossed and re-crossed before the mental status becomes finally certain. Delusive ideas of to-day may be dissipated on the morrow, only to be renewed with greater force and more full control, till at last they are received with a full belief in their reality. It is during this state of uncertainty and doubt that not unfrequently the person has an appreciation of his condition, realizes the fact that he is becoming insane, and seeks relief from his trouble. This period promises the best and speediest results to judicious medical treatment, and hence the importance of its full appreciation by the physician.

"With the advance of medical science, insanity has been more and more practically understood; and, happily, the mystery and dread associated with it melt away under the light of investigation and experience; and both physicians and laymen see it in a less formidable light, when it is brought before them in its true character of a cerebral disease, and only a disease. Now the triumph is complete, mystery and superstition vanish, and the insane man stands forth simply as a sick man: one, who by reason of cerebral disease, is unable to use his brain—not a man with a mind diseased, a mad mind, an enfeebled mind—but with a brain and nervous system so disordered as to disturb, confuse, heighten, or lessen the mental operations; a mind acting through a disordered organ—a spiritual being untouched by disease, looking through the disordered and broken house in which he dwells."*

———:o:———

An argument in favor of compulsory vaccination is found in the fact that in Philadelphia, with nearly a million inhabitants, but where vaccination is general, there was not a single death last year from small-pox. Under the old *régime* the number of deaths should be at least a thousand annually, which would be greatly multiplied during an epidemic.

* Transactions of New York State Medical Society for 1868, page 88, op. cit.

